# Cryo-EM structure of the large subunit of the spinach chloroplast ribosome

**DOI:** 10.1038/srep35793

**Published:** 2016-10-20

**Authors:** Tofayel Ahmed, Zhan Yin, Shashi Bhushan

**Affiliations:** 1 School of Biological Sciences, Nanyang Technological University, Singapore; 2 NTU Institute of Structural Biology, Nanyang Technological University, Singapore

## Abstract

Protein synthesis in the chloroplast is mediated by the chloroplast ribosome (chloro-ribosome). Overall architecture of the chloro-ribosome is considerably similar to the *Escherichia coli* (*E. coli)* ribosome but certain differences are evident. The chloro-ribosome proteins are generally larger because of the presence of chloroplast-specific extensions in their N- and C-termini. The chloro-ribosome harbours six plastid-specific ribosomal proteins (PSRPs); four in the small subunit and two in the large subunit. Deletions and insertions occur throughout the rRNA sequence of the chloro-ribosome (except for the conserved peptidyl transferase center region) but the overall length of the rRNAs do not change significantly, compared to the *E. coli*. Although, recent advancements in cryo-electron microscopy (cryo-EM) have provided detailed high-resolution structures of ribosomes from many different sources, a high-resolution structure of the chloro-ribosome is still lacking. Here, we present a cryo-EM structure of the large subunit of the chloro-ribosome from spinach (*Spinacia oleracea*) at an average resolution of 3.5 Å. High-resolution map enabled us to localize and model chloro-ribosome proteins, chloroplast-specific protein extensions, two PSRPs (PSRP5 and 6) and three rRNA molecules present in the chloro-ribosome. Although comparable to *E. coli*, the polypeptide tunnel and the tunnel exit site show chloroplast-specific features.

Protein translation is a universal process of amino acid polymerization mediated by the ribonucleoprotein complex called the ribosome^1^,^2^. The ribosomes are composed of two different subunits, called the large subunit (LSU) and the small subunit (SSU). The SSU is mainly responsible for decoding of mRNA by selecting the correct charged tRNAs (cognate tRNAs)^1^,^3^. The LSU catalyzes amino acid polymerization (peptide bond formation)^4^,^5^ and provides a tunnel through which nascent polypeptides leave the ribosome. Exit site of the tunnel provide a platform for binding of various factors required for processing, targeting and membrane insertion of the nascent polypeptides^5^,^6^,^7^. In *Escherichia coli* (*E. coli*) 70S, the SSU is composed of 16S rRNA and 21 ribosomal proteins while the LSU consists of 5S and 23S rRNA along with 33 ribosomal proteins. The eukaryotic cytoplasmic ribosome (80S) is larger in size than the bacterial 70S ribosome, as it contains more proteins and rRNAs^8^,^9^.

The mitochondria and chloroplasts carry their own genome. Genes encoded by these organellar genomes are transcribed by their own transcriptional machineries and translated by the unique ribosomes present in these organelles^10^,^11^. The mitochondrial ribosome (mitoribosome) has evolved significantly in terms of composition^11^, structure and function^12^ in comparison to their bacterial ancestors. The mitoribosomes contain a number of mitochondria-specific proteins and protein extensions^13^,^14^,^15^,^16^ and the rRNA content is significantly different both in the composition and size^17^,^18^,^19^,^20^. These structural changes have enabled the mitoribosome to adapt in mitochondrial environment and specialize in synthesizing mainly membrane proteins of respiratory chain complexes^21^. Recent high-resolution cryo-electron microscopy (cryo-EM) structures of the mitoribosomes^22^,^23^,^24^,^25^,^26^,^27^ have provided unparalleled details about the structure and function of these unique ribosomes.

According to the endosymbiotic theory^28^, the chloroplast originated from a free-living photosynthetic prokaryote. During evolution, most of the chloroplast genes were transferred to the nucleus^29^. The chloro-ribosome synthesizes only a number (~6–7%) of the proteins functioning inside the chloroplast and majority of them are post-translationally imported from the cytoplasm^30^,^31^,^32^,^33^. The components of some of the protein complexes are encoded by both the chloroplast and nuclear genome^34^,^35^ in many instances and therefore, it is no surprise that the gene expression between these two organelles is tightly regulated^30^,^36^,^37^. This is to maintain the stoichiometry of the protein subunits present in these complexes. The light, which changes the redox states of the components involved in photosynthetic electron transport chain, is a major signal controlling the regulation of protein expression in the chloroplast^33^,^38^. A total of 146 genes have been identified in the circular genome of the spinach (*Spinacia oleracea*) chloroplast out of which 98 encodes for proteins^31^. Majority of these proteins form subunits of the multimeric membrane protein complexes such as ATP synthase, photosystem-1 and −2, cytochrome *b6/f* complex and RuBisCO (the large subunit), all of which are involved either in photosynthesis or respiration. Other examples of chloroplast-encoded proteins are RNA polymerases, NADH dehydrogenases, ribosomal proteins and translation factors^31^,^33^. In general, the chloro-ribosome translates more proteins than the mitoribosome. Unlike the mitoribosome, which mainly synthesizes hydrophobic membrane proteins^21^,^39^, the chloro-ribosome synthesizes both soluble and membrane proteins^10^,^40^. This can be one of the reasons that chloro-ribosomes have not changed as much as mitoribosomes in comparison to their bacterial ancestor.

The overall composition of the chloro-ribosome is similar to the *E. coli* 70S but differences occur^30^,^31^. Most of the chloro-ribosome proteins (also known as plastid ribosomal proteins or PRPs) are longer due to the presence of chloroplast-specific extensions at their N- and C-termini^31^. Compared to the *E. coli* 70S, two of the LSU proteins (bL25 and uL30) are absent and six new plastid-specific ribosomal proteins (PSRPs) are added: four in the SSU and two in the LSU^31^,^40^. Deletions and insertions are observed throughout the rRNA sequence except for the conserved peptidyl transferase centre (PTC) region^31^. Two earlier cryo-EM structures of chloro-ribosomes at 15.5^30^ and 9.4 Å^31^ resolution provided the first glimpse of their structures. However, molecular insights into the structure of the chloro-ribosome is still lacking due to unavailability of a high-resolution structure.

Here, we report a near-atomic resolution structure of the chloro-ribosome LSU from spinach at 3.5 Å resolution determined using cryo-EM and single particle analysis. High-resolution map allowed *de novo* modelling of two PSRPs, many of the protein extensions and rRNA insertions. The overall structure resembles the *E. coli* ribosome LSU but chloroplast-specific features are visible. PTC is conserved but noticeable differences in the structure of proteins lining the polypeptide tunnel and tunnel exit site are present. Our study aims to fill a gap in the field of high-resolution structures of the ribosome available and reveals unique features of the chloro-ribosome at near-atomic resolution.

## Results and Discussion

### Structure determination of spinach chloro-ribosome LSU

**Figure 1:**
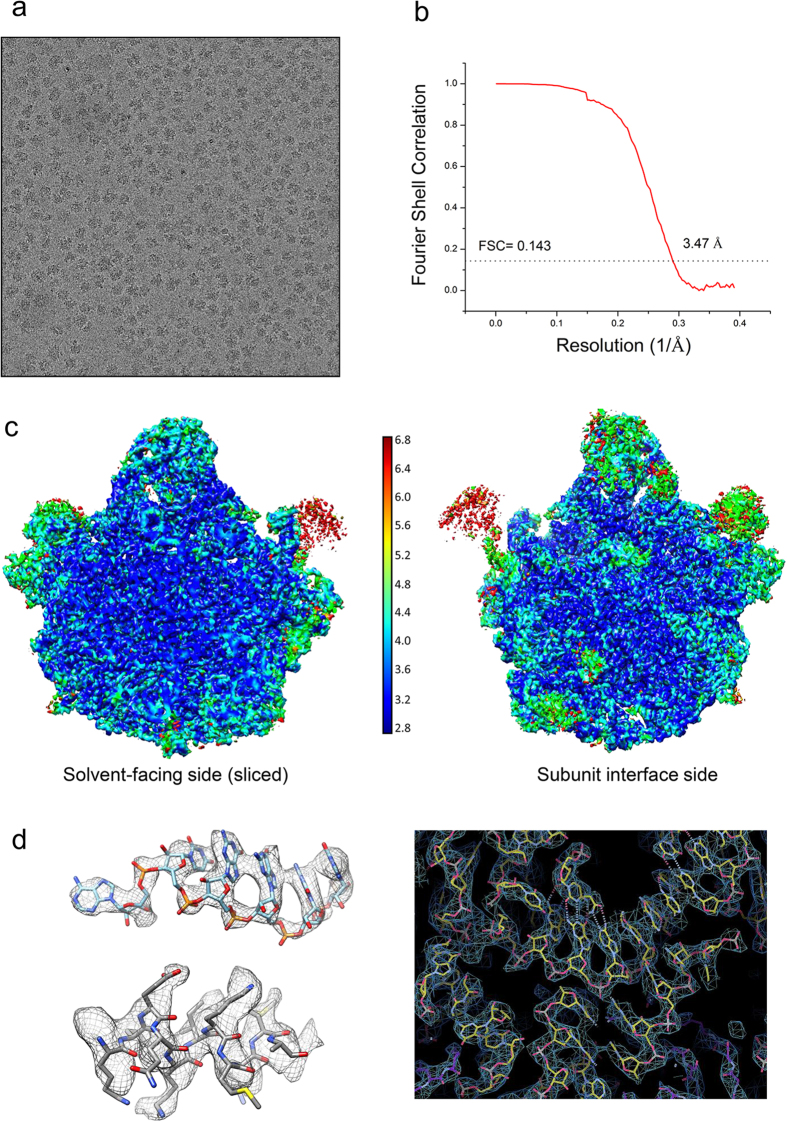
Processing of cryo-EM data. (**a**) Representative micrograph showing ribosome particles. **(b)** Fourier Shell Correlation (FSC)^61^ curve showing the average resolution of the reconstructed density map of the spinach chloro-ribosome LSU. **(c)** Local resolution estimation of cryo-EM map by ResMap^42^. Map is coloured according to local resolution of masked map. **(d)** Representative density showing a 23S rRNA region (nucleotides 2584–2589) in upper left, a helix of PSRP5 protein (amino acid residues 99–109) in lower left and a region around nucleotide G2462 in 23S rRNA on the right, showing quality of the map and fitting of the models.

**Figure 2:**
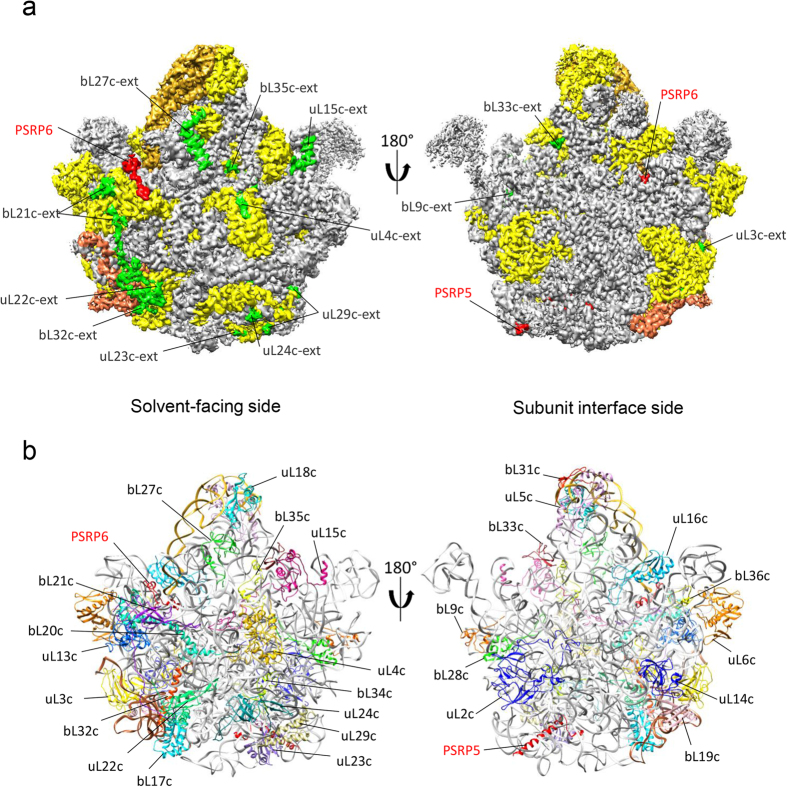
Overview of the spinach chloro-ribosome LSU. **(a)** Cryo-EM reconstructed density of the spinach chloro-ribosome LSU, showing solvent-facing and subunit interface side. All proteins except two PSRPs (red) are coloured yellow. Chloroplast-specific extensions are coloured green. rRNAs are coloured as: 5S (goldenrod), 4.8S (sienna) and 23S (grey). **(b)** Overview of the proteins and rRNAs modelled in the spinach chloro-ribosome LSU density showing solvent-facing (left) and subunit interface side (right). The protein and rRNA models are differently coloured.

**Figure 3:**
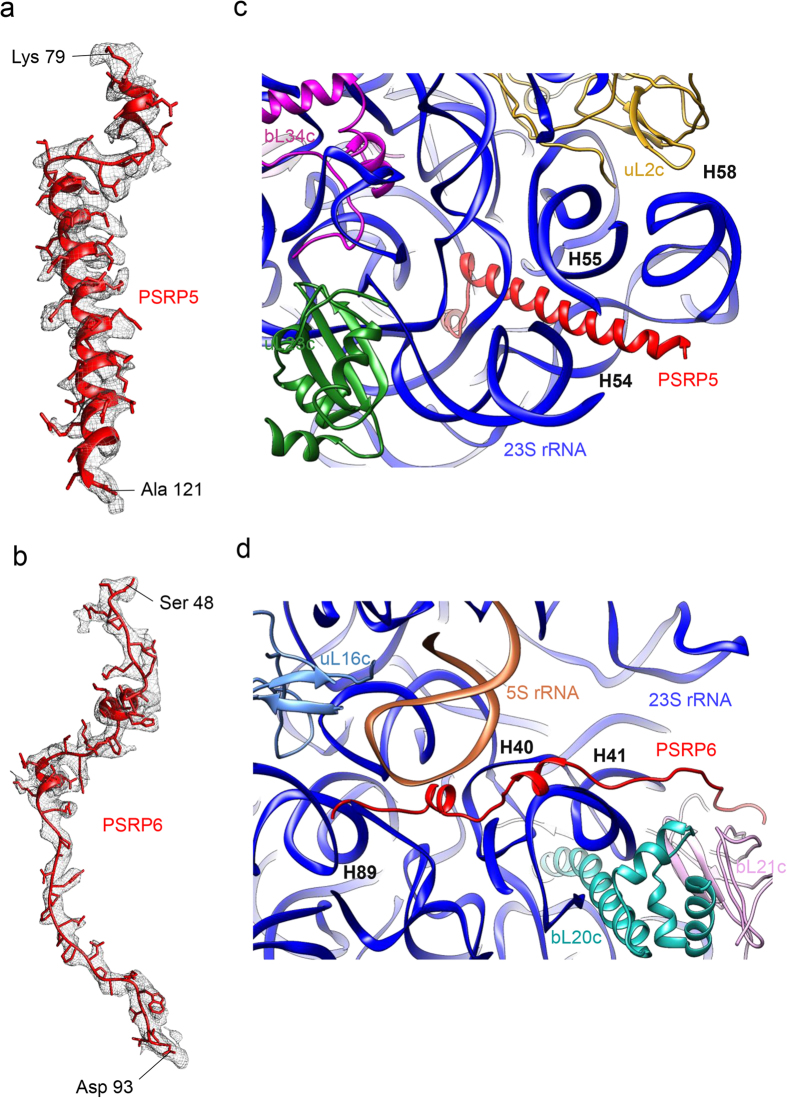
Locations and structures of PSRP5 and PSRP6. (**a,b**) Models of the PSRP5 (**a**) and PSRP6 (**b**) showing side chains fitted inside density. (**c**) Location of PSRP5 in an all-rRNA environment. (**d**) Location of PSRP6 in close association with rRNA and bL21c.

Chloro-ribosomes were purified from spinach leaves using sucrose density gradient centrifugation according to the published procedures^31^,^41^. Cryo-EM and single particle analysis were employed to determine the structure of the chloro-ribosome. A representative electron micrograph of the purified chloro-ribosome is shown in Fig. 1a. Attempts to determine a high-resolution structure of the complete chloro-ribosome (70S) were unsuccessful because of the over representation of the 50S particles in our dataset which might have resulted due to subunit dissociation during purification (as revealed by 2D and 3D classification, Supplementary Fig. S1). Therefore, we focused on determining the structure of the chloro-ribosome LSU using masked refinement procedures^27^. A soft Gaussian mask was created from a 3D reconstruction of the chloro-ribosome LSU generated during 3D classification step (Supplementary Fig. S1). The cryo-EM and single particle reconstruction using this focused mask during 3D refinement step resulted in a cryo-EM density map of the chloroplast LSU at an average resolution of 3.5 Å (Fig. 1b), while best resolved regions of the map contained information up to 2.8 Å resolution as suggested by the local resolution estimation with ResMap^42^ (Fig. 1c). Most of the protein side chains and rRNA bases were resolved in our map (Fig. 1d), which enabled us to build a near-atomic-resolution model of the spinach chloro-ribosome LSU. Our model comprises of 28 *E. coli* orthologous proteins (Fig. 2b) with many containing chloroplast-specific N- and C- terminal extensions (Fig. 2a and Supplementary Fig. S2), two PSRPs (Figs 2 and 3) and 4.8S, 5S and 23S rRNAs (Fig. 2). Both the L1 and L7/L12 stalk regions have weak densities in the map due to flexibility and therefore models for the proteins- uL1c, uL10c, uL11c and bL12c could not be built.

### Overall structure of spinach chloro-ribosome LSU

Since the chloro-ribosome is more similar to the *E. coli* ribosome than the mitoribosome^30^,^31^, we compared our map of the spinach chloro-ribosome LSU to the *E. coli* 50S ribosome. Atomic model of the *E. coli* 50S (PDB ID: 4YBB)^43^ fitted very well in our map (Supplementary Fig. S3) and therefore was used as a starting point to generate models for the spinach chloro-ribosome LSU. Rigid body docking of the *E. coli* ribosome 50S model into our map lead to the identification and localization of the densities for the *E. coli* orthologous proteins and rRNAs present in the spinach chloro-ribosome LSU. A combination of secondary structure prediction, homology modelling and tracing of amino acid side chains were used for model building. Details of the protein models are presented in Table 1.

**Figure 4:**
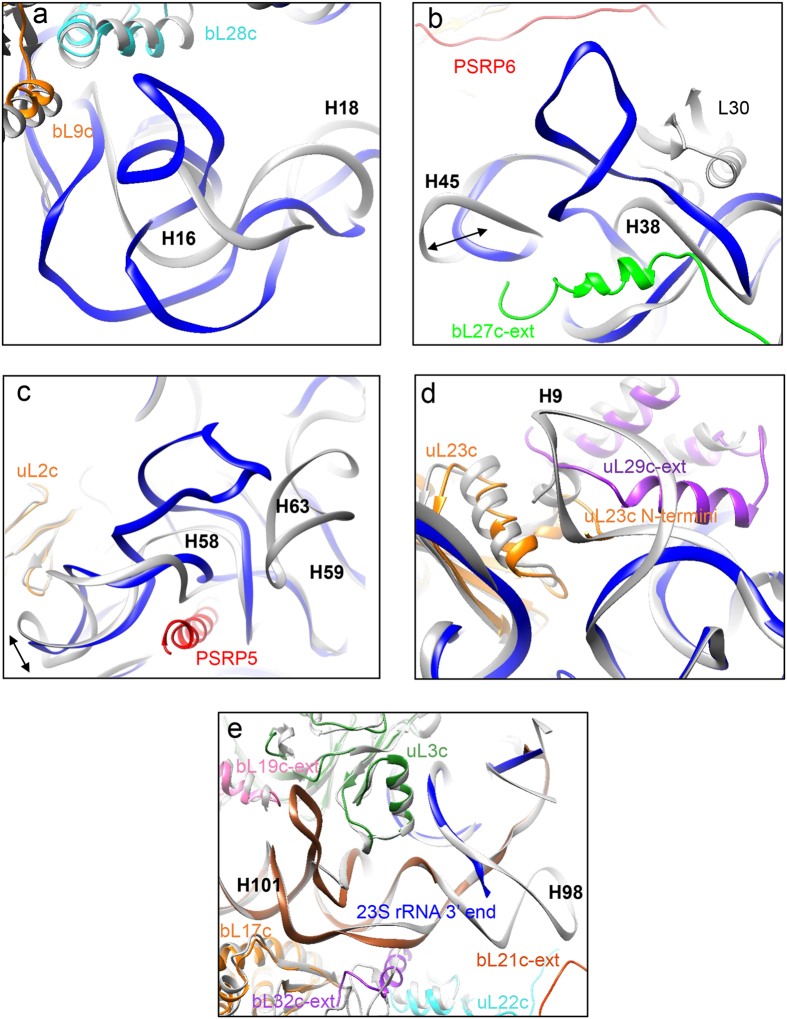
Remodelled rRNAs of the spinach chloro-ribosome LSU. Regions in the 23S rRNA (**a,d**) and 4.8S rRNA (**e**) which have been remodelled because of rRNA insertions or deletions. Models for the spinach chloro-ribosome LSU (built in the current study) and the *E. coli* (PDB ID: 4YBB) are rigid body fitted into the cryo-EM map of the spinach chloro-ribosome LSU for comparison. The 23S rRNA of the spinach chloro-ribosome LSU is shown in blue in all cases and the chloro-ribosome LSU proteins are coloured and labeled. 4.8S rRNA (**e**) is shown in sienna. Overlaid *E. coli* models are indicated in grey.

All protein orthologues of the *E. coli* ribosome LSU could be identified in the spinach chloroplast LSU map with the exceptions of two proteins, namely bL25 and uL30 (Supplementary Fig. S3). This is consistent with earlier proteomic studies^40^ where both the bL25 and uL30 proteins could not be identified in the chloro-ribosome. Interestingly, two novel PSRPs (PSRP5 and PSRP6) have been acquired, keeping the total number of proteins in the LSU of spinach chloro-ribosome similar to the *E. coli* LSU. In comparison to the *E. coli* ribosome LSU, which contains two rRNA molecules (5S and 23S), the spinach chloro-ribosome LSU contains an additional rRNA molecule named as 4.8S rRNA (Supplementary Fig. S3). Chloroplast-specific N- and C-terminal extensions are located at the solvent exposed side and the two PSRPs are present at novel sites mainly surrounded by rRNA (Figs 2 and 3). Subunit interface side is almost unchanged suggesting a conserved PTC (Fig. 2). Major alterations and remodelling of the rRNAs occur at their flexible end towards the periphery (Fig. 4, Supplementary Figs S4 and S5).

### Chloroplast-specific protein-extensions

Although the total number of proteins present in the spinach chloro-ribosome LSU is same as in the *E. coli* 50S ribosome^8^,^40^, chloro-ribosome proteins are usually longer in the length. The increment in length is due to the presence of the chloroplast-specific protein extensions at their N- and C-termini. Among the 31 *E. coli* orthologous proteins present in the spinach chloro-ribosome LSU, 25 contain chloroplast-specific extensions which are variable in their sizes, ranging from as small as 3 residues (in uL18c) to as long as 97 residues (in bL21c). We have localized and partially modelled 16 of these protein extensions, *de novo* (Supplementary Fig. S2). These extensions are mostly located on the solvent-exposed side of the chloro-ribosome LSU (Fig. 2). Secondary structural elements can be identified in some of the extensions but in most cases extensions maintain an extended conformation which resulted in poorly resolved densities and therefore complete structural modelling of all the extensions could not be possible.

A summary of the modelled chloroplast-specific extensions is presented in Supplementary Table S1. bL21c and uL22c proteins are almost doubled in size compared to their *E. coli* orthologues because of their N- and C-terminal extensions. The combined extensions at both the N- and C-termini, which are longer than 20 residues in length, are present in L1 (46 residues), L4 (39 residues), L5 (41 residues), L13 (49 residues), L15 (37 residues), L19 (46 residues), L21 (97 residues), L22 (89 residues), L23 (35 residues), L24 (40 residues), L27 (53 residues), L29 (46 residues), L31 (23 residues) and L32 (23 residues). The presence of these extensions in the chloro-ribosome proteins are mainly responsible for altered protein: rRNA ratio in the chloro-ribosome (2:3) as compared to the *E. coli* (1:3)^31^,^40^. Notably, some of these extensions are located in positions where alterations in rRNA structures are visible and are discussed in details in the following section. The close association of these extensions with rRNA is suggestive of their possible role in maintaining the structural integrity of the chloro-ribosome.

### Structures of PSRP5 and PSRP6

After assigning the densities for all of the *E. coli* orthologous proteins (except proteins residing in the L1 and L7/L12 stalks), chloroplast-specific protein extensions and rRNAs, extra densities at two places remain unaccounted. High-resolution map allowed us to identify the unaccounted densities as PSRP5 and PSRP6 and build *de novo* models for these two proteins (Fig. 3).

PSRP5 is a conserved, nuclear-encoded, plastid-specific protein of 142 amino acids in length (mature protein consists of 80 aa) and basic in nature. As expected from the basic nature of PSRP5, its density is surrounded by rRNA suggesting its close association with rRNA (Fig. 3c). Density for PSRP5 residues ranging from 79 to 121 is clearly visible in our map (Fig. 3a) while densities for the first 16 N-terminal and the last 21 C-terminal residues are missing possibly because of their flexibility. The density for the residues ranging from 92–121 is seen to adopt a helical conformation while residues ranging from 80–91 remains partially disordered with the presence of a small alpha-helical twist between residues 80–85 (Fig. 3a). Structure of the rRNA around PSRP5 is remodelled (discussed in details in the next section) in the chloro-ribosome as compared to the *E. coli* 50S. Therefore, it seems plausible to hypothesize that chloroplast has specifically evolved to incorporate PSRP5 in their ribosomes to stabilize the altered rRNA structure around its position.

Localization of the PSRP5 in our map does not agree with Sharma *et al*.^31^, who had earlier localized PSRP5 near to the tRNA exit site (E-site). Analysis of our map suggests the presence of the chloroplast-specific C-terminal extension of the protein uL15c in this region (Fig. 2b). Mutations in the psrp5 gene have been reported to impair growth and reduce photosynthesis in Arabidopsis^44^. Subunit accumulation studies using Arabidopsis psrp5 mutants have suggested that PSRP5 might be required for the assembly and stability of the chloro-ribosome LSU^44^. The close association of PSRP5 with rRNA, as observed in our structure, raises such a possibility. However, the RNA-buried appearance of PSRP5 in our map indicates that it might not be involved in light-dependent regulation of translation in chloroplast as speculated earlier^30^,^31^.

PSRP6, the second of the PSRPs present in the spinach chloro-ribosome LSU, is also a conserved, nuclear-encoded, plastid-specific protein of 116 amino acids in length (mature protein consists of 69 aa) and basic in nature. However, in contrast to the PSRP5, density for the PSRP6 is mainly extended with the presence of minimal secondary structural features (Fig. 3b). The density for the residues ranging from 48 to 93 is clearly visible in the map whereas the density for the last 23 C-terminal residues is missing probably due to flexibility. The local environment of the PSRP6 in the LSU is shown in Fig. 3d. The N-terminal of PSRP6 is buried between a groove formed by 5S rRNA and H89 of 23S rRNA while C-terminal extends towards protein bL21c present on the surface of the LSU. Our *de novo* modelling of the PSRP6 reveals that it is mostly disordered except for two very short stretches (residues ranging from 57 to 62 and 69 to 73) of helical turns in the N-terminal region. Interestingly, in the previous cryo-EM study of spinach chloro-ribosome^31^, authors could not identify PSRP6 due to the low-resolution of their map and suggested that it might be relatively loosely associated with the ribosome. However, our map suggests that PSRP6 is an integral part of the spinach chloro-ribosome LSU. It has been reported that PSRP6 is dispensable for chloroplast ribosomal assembly and function under normal growth conditions^44^. However, it is possible that PSRP6 is required under unfavorable growth conditions. An interesting feature of the PSRP6 is the presence of a proline-rich motif (residues 80 to 86) which is located at the solvent exposed side of the LSU. Proline-rich motifs have earlier been suggested to act as ligands of modulator proteins in the eukaryotic signal transduction pathways^45^. Therefore, it is possible that the proline-rich motif present in PSRP6 is used as an accessible site for the non-ribosomal factors to interact with the chloro-ribosome^45^. The surface exposed presence of this motif in PSRP6 is reminiscent of such a possibility.

The fact that both bL25 and uL30 are absent from the chloro-ribosome LSU while two new proteins, namely PSRP5 and PSRP6 have been acquired, lead to the assumption that these two PSRPs might have replaced the role of bL25 and uL30 in the chloro-ribosome. However, locations and structures (Fig. 3 and Supplementary Fig. S3) of these two PSRPs suggest that they are not a substitution of bL25 and uL30 and might be required for some other specialized functions during ribosome assembly or protein synthesis in the chloroplast.

### Remodelling of rRNA structures

Total length of the rRNA in the spinach chloro-ribosome LSU is 3037 nucleotides, which is 13 nucleotides longer than the *E. coli* 50S rRNA (3024 nt). Unlike *E. coli* 50S, where only 5S (120 nt) and 23S rRNAs (2904 nt) are present, chloro-ribosomes contain three molecules of rRNAs named as 4.8S (106 nt), 5S (121 nt) and 23S rRNA (2810 nt).

Global sequence alignment using EMBOSS^46^ needle tool reveals that 23S rRNA is 64.7% and 5S rRNA is 58.7% identical in sequence between the *E. coli* LSU and the spinach chloro-ribosome LSU. Nucleotide sequence alignment also reveals that insertions and deletions have occurred in the entire span of rRNA sequence in chloro-ribosome. But deletion in a position is balanced by insertion in a nearby position (and vice versa), keeping the overall structure of the chloro-ribosome rRNA comparable to the *E. coli*. However, analysis of our map reveals that nucleotide insertions and deletions have altered local rRNA structure significantly at five different places (Fig. 4 and Supplementary Figs S4 and S5). Four of these regions are located in 23S rRNA surrounding nucleotide positions 280–315, 939–968, 1502–1526 (chloroplast 23S rRNA numbering) and 129–153 (*E. coli* 23S rRNA numbering) and the fifth one is located in the 4.8S rRNA surrounding positions 1–28. All of these alterations in the rRNA structure are located far from the conserved core (PTC forming region of domain V and VI in 23S rRNA).

The first region with alterations in rRNA structure results because of a combined 24 nucleotides long insertion in domain I of 23S rRNA between positions 280–315, involving H18 (uppercase H is used to denote rRNA helices and numbering of rRNA helices are based on *E. coli* 23S rRNA secondary structure diagram obtained from http://rna.ucsc.edu/rnacenter/ribosome_images.html) and H19 (Fig. 4a and Supplementary Figs S4a and S5a). Insertion in this region does not change the structure of H18 and H19 but causes a slight shift in the axis of H18. Surprisingly, topology of neighbouring H16 is perturbed, which forms a variable loop structure and extends further towards the solvent side (Fig. 4a and Supplementary Figs S4a and S5a).

The second region with alterations in rRNA structure results due to a combined 20 nucleotides long insertion in domain II of 23S rRNA between positions 938–969 (H38, Fig. 4b and Supplementary Figs S4b and S5b). This insertion remodels H38 by introducing a protrusion which extends towards the solvent side. Interestingly, this protrusion is located near to a place where uL30 is situated in the *E. coli* ribosome LSU. Because uL30 is absent in the chloro-ribosome, it is possible that the function of uL30 is being compensated by this variable loop of rRNA. Interestingly, PSRP6 and chloro-specific C-terminal extension of bL27c are present in close vicinity of this protrusion. Variability around this region also includes shortening (indicated by an arrow) of H45 due to a net deletion of 6 nucleotides in the chloro-ribosome 23S rRNA sequence.

The third region with alterations in the rRNA structure results because of a combined 12 nucleotides long insertion in domain III of 23S rRNA between positions 1502–1526 (H58). This insertion creates a protrusion in H58 which extends towards the solvent exposed side (Fig. 4c and Supplementary Figs S4c and S5c left panel). Local changes resulting from this insertion event have remodelled the rRNA causing shortening (indicated by an arrow) of H58 which is located towards uL2c. In *E. coli* ribosome, H63 extends near to this region but deletion of 26 residues in equivalent position in chloroplast 23S rRNA sequence makes H63 much shorter (Fig. 4c and Supplementary Figs S4c and S5c right panel).

The fourth region with alterations in rRNA structure results because of a combined 14 nucleotides long deletion in domain I (*E. coli* numbering 129–153) of 23S rRNA and results in the deletion of H9 from the chloroplast 23S rRNA structure (Fig. 4d and Supplementary Figs S4d and S5d). Presence of chloroplast-specific C-terminal extension of uL29c around this region suggest a possible replacement of *E. coli* H9 with this chloro-specific protein extension.

The last region with significant alterations in rRNA structure results from a deletion and an insertion in the 4.8S rRNA (Fig. 4e and Supplementary Figs S4e and S5e). Deletion of a continuous stretch of 16 nucleotides at the 5′ end of 4.8S rRNA results in the absence of H98 in the spinach chloro-ribosome LSU. Interestingly, this deletion shares the same region where presumably 4.8S rRNA had got cleaved from its precursor 23S rRNA, and evolved as a new rRNA molecule. A 9 nucleotides long insertion at position 28 in chloro 4.8S rRNA (domain VI in the *E. coli* 23s rRNA) creates a bulge between H100 and H101, which resides in close proximity with chloro-specific N-terminal extension of bL19c.

Sequence analysis indicates that 4.8S rRNA is similar to the 3′ end of the *E. coli* 23S rRNA. Therefore, it is possible that 4.8S rRNA might have got separated from a 23S rRNA precursor during evolution and thereafter co-existed as a separate rRNA molecule along with the 23S rRNA to facilitate rRNA remodelling by loosing H98 at this cleavage site (Fig. 4e and Supplementary Figs S4e and S5e). The loss of H98 might have been necessary for the structure or function of the chloro-ribosome. Detachment of 4.8S rRNA from 23S rRNA makes chloroplast 23S rRNA shorter in length by 94 nucleotides compared to the *E. coli* 23S rRNA.

H38 and H58 in the 50S subunit of *H. marismortui* ribosome have earlier been reported as Kink-turn (K-turn) containing sites by Klein *et al*.^47^. K-turns are characterized by the presence of the helix-turn-helix structure, which help in introducing flexibility in rRNA structures^48^. K-turns act as important rRNA recognition motifs for the ribosomal proteins and form nucleation points during ribosome assembly. Introduction of the protrusions in chloro-ribosome 23S rRNA (H38 and H58) near to the positions where K-turns are present in *H. marismortui* 23S rRNA (Supplementary Fig. S6) is an interesting feature of these ribosomes that might have structural or functional implications in chloro-ribosomes.

### Polypeptide tunnel and tunnel exit site

**Figure 5:**
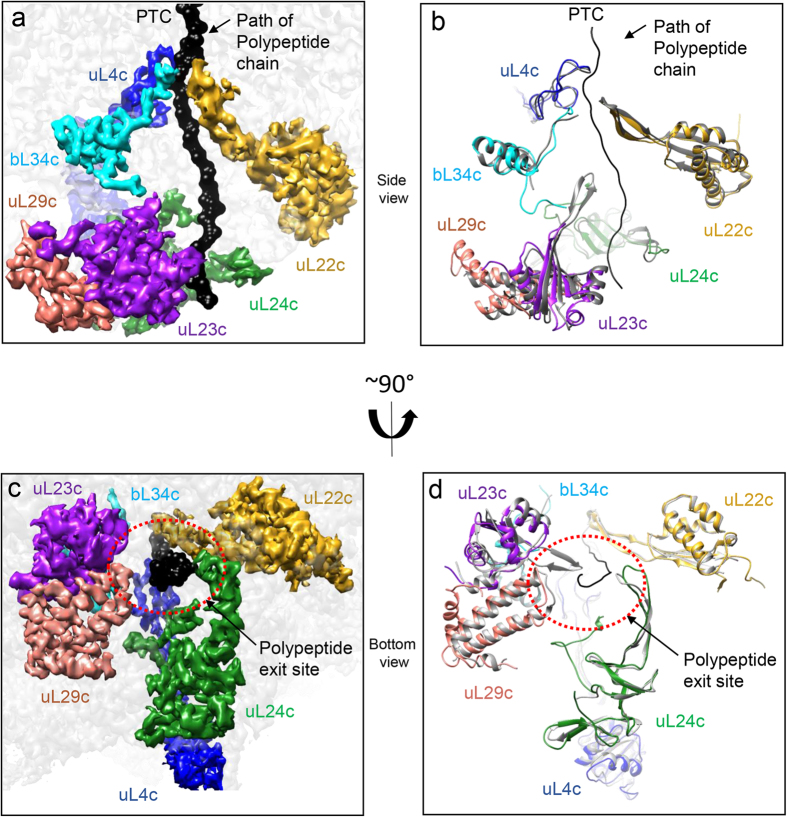
The polypeptide exit tunnel and polypeptide tunnel exit site of the spinach chloro-ribosome LSU. (**a**) Polypeptide exit tunnel of the spinach chloro-ribosome indicated by the path of the simulated nascent chain (in black surface). Densities for the proteins lining the tunnel wall are isolated and shown in surface as indicated. (**b**) Models of the chloro-ribosome proteins lining the tunnel wall are overlaid with the *E. coli* protein models to show the chloro-specific features of the exit tunnel. (**c**) View as in (**a**) is rotated to show the polypeptide tunnel exit site in the spinach chloro-ribosome LSU. (**d**) Rotated view of (**b**) showing chloro-specific features of the polypeptide tunnel exit site in comparison to the *E. coli* tunnel exit site. Protein models and densities are labeled as indicated. For clarity rRNA is omitted.

Nascent polypeptide chains travel through a polypeptide tunnel of about 100 Å in the LSU and interact with a diverse range of protein biogenesis factors involved in protein maturation, folding and targeting at the tunnel exit site. The tunnel exit site acts as a docking platform for these protein biogenesis factors. In *E. coli* ribosomes, polypeptide exit tunnel is mainly consisted of 23S rRNA along with three ribosomal proteins (uL4, uL22 and uL23) while the tunnel exit site is surrounded by uL22, uL23, uL24 and uL29. Our map of the spinach chloro-ribosome LSU reveals that the overall architecture of the polypeptide tunnel and tunnel exit site is similar to the *E. coli* 50S. However, certain differences are visible. The density for a conserved beta hairpin loop present in the *E. coli* uL23, which extends to the polypeptide tunnel is absent in uL23c (Fig. 5 and Supplementary Fig. S7). Additionally, N-termini of uL23c is mainly disordered and extends towards a position where H9 (absent in the chloro-ribosome) is located in the *E. coli* 50S (Fig. 4d). Moreover, C-termini of uL23c adopts a small helical turn while, it is in extended conformation in *E. coli* L23 (Supplementary Fig. S7). uL23 has been proposed to act as a universal docking site^49^ for a number of nascent polypeptide processing factors such as the signal recognition particle (SRP)^50^, trigger factor (TF)^50^,^51^ and SecA^52^, etc. The Absence of this loop in the chloro-ribosome makes the tunnel wider at this position. In *E. coli*, this loop extends into the polypeptide tunnel to form a potential interaction site with the nascent chain. Truncation of parts of the beta hairpin loop in *E. coli* does not impair cell growth under normal growth conditions but has been shown to lower the affinity of SRP binding to the ribosome nascent chain complex (RNC)^53^. It has also been proposed that the interaction between the nascent chain and this beta hairpin loop in the tunnel induces conformational change in L23 at the tunnel exit site, therefore increasing the affinity of SRP to bacterial RNC^53^. The SRP system in the chloroplast is unique in both the composition and function^54^,^55^. In chloroplast, the RNA component of *E. coli* SRP (4.8S RNA) is substituted with a protein named as cpSRP43. Unlike the *E. coli* SRP system which functions only co-translationally, the cpSRP has been suggested to function both co-translationally and post-translationally. The alteration in the structure of uL23c raises a possibility that a different mechanism of SRP recruitment is followed by the chloro-ribosome. However, further investigations are required to confirm this hypothesis.

The density for 15 amino acids of the chloroplast-specific N-terminal extension of uL24c is clearly visible in our map which extends towards the polypeptide tunnel suggesting a possible chloroplast-specific interaction between uL24c and nascent polypeptide (Fig. 5). Density for 24 amino acids of the chloroplast-specific C-terminal extension of uL29c can also be clearly seen in the map. This extension is located parallel to the N-terminal extension of uL23c. In the C-terminal extension of uL29c, the residues spanning 130 to 141 adopt a helical structure, while the residues ranging from 142 to 149 are seen to be extended in conformation. Taken together, it can be postulated that the significant differences present in three (uL23c, uL24c and uL29c) out of the four proteins (uL22c, uL23c, uL24c and uL29c) present at the tunnel exit site might reflect a chloroplast-specific mode of recruitment of protein biogenesis factors on the chloro-ribosome.

## Methods

### Purification of chloro-ribosomes

Purification of chloro-ribosomes was performed as previously described^31^,^41^, with some modifications in the protocols. Briefly, six kilograms of baby spinach (*Spinacia oleracea*) leaves bought from the local market were deveined, washed (using distilled water) and homogenized using 0.7 M sorbitol in buffer A (10 mM Tris·HCl, pH 7.6, 50 mM KCl, 10 mM MgOAc, and 7 mM 2-mercaptoethanol) using a blender (two shots of 10 sec each). The homogenized leaves were filtered through a double-layer of cheesecloth followed by one layer of Miracloth (Calbiochem). Resultant filtrate was centrifuged at 1200 × g for 15 min. The pelleted chloroplast was resuspended in 0.4 M sorbitol in buffer A and thereafter centrifuged again at 1200 × g for 15 min. The pellet was resuspended in 2% (vol/vol) Triton X-100 in buffer A and incubated on ice for 30 min. The lysed suspension was centrifuged at 26,000 × g for 30 min to achieve clarification and thereafter crude ribosomes were pelleted by centrifugation at 86,000 × g for 17 hr through a sucrose cushion (1 M sucrose in buffer A). The pellet was washed and gently resuspended in buffer B (buffer A with 10% glycerol). The suspension (crude ribosomes) was clarified by centrifugation at 26,000 × g for 15 min and was loaded onto 10–40% sucrose gradient in buffer A (with RNase inhibitor) and centrifuged at 111,000 × g for 4 hr. Fraction corresponding to 70S chloro-ribosomes was collected and further centrifuged through a sucrose cushion (0.75 M sucrose in buffer A, with RNase inhibitor) at 84,000 × g for 2.5 hr. The chloro-ribosome pellet was suspended in a grid buffer containing 20 mM Tris·HCl, pH 7.6, 100 mM KCl, 10 mM MgOAc, 100 mM sucrose, 7 mM 2-mercaptoethanol, 1 unit/ml RNase inhibitor and 0.1% protease inhibitor.

### Electron microscopy

4 μl of purified chloro-ribosome at final concentrations of 4 OD_260_/ml were applied onto glow-discharged 2-nm carbon coated holey grids (Quantifoil R2/2) and incubated for 30 sec. Grids were blotted for 3 sec in 100% humidity at 4 °C and flash frozen in liquid N_2_-cooled liquid ethane using FEI Vitrobot. Grids were loaded to an FEI Tecnai Arctica cryo-transmission electron microscope, operated at 200 kV and equipped with a back-thinned Falcon III direct electron detection device. Electron micrographs were collected automatically by using FEI’s automatic single particle acquisition software EPU. A total of 1,729 micrographs were recorded in a movie mode as a set of 7 frames (total dose of 26 electrons per Å^2^) at a calibrated magnification of 109,375 resulting in a pixel size of 1.28 Å on the object scale and at defocus values from 0.2–2.5 μm.

### Image processing

After data collection and screening for good micrographs, 1590 micrographs were selected for data processing. Whole-image drift correction was carried out using motioncorr^56^. e2boxer.py from EMAN 2.1^57^ was used to semi-automatically pick particles from motion-corrected micrographs and CTF parameters were estimated using CTFFIND3^58^. Data was processed with RELION 1.4^59^. In total, 338,305 particles were subjected to reference-free 2D classification to discard bad particles. 261,052 particles selected from the 2D classes were directly used for 3D refinement using a 60 Å filtered *E. coli* 70S map^60^ and thereafter 3D classified without image alignment. 3D refinement of the particles from the 3D classes representing 70S particles did not yield a high-resolution map for the complete chloro-ribosome most probably because of the number of the particles being limited and the presence of conformational heterogeneity between the ribosomal subunits. Therefore, particles from both the 70S and 50S 3D classes were combined and further processed using a soft mask on the 50S subunit (Supplementary Fig. S1) to obtain a high-resolution map of the LSU. 202,766 particles from the high-resolution 3D classes obtained with a 50S mask were sorted according to the defocus values of the micrographs. 3D refinement of 174,949 particles with defocus values lower than 1.5 μm yielded a 50S density map at 3.5 Å resolution (Supplementary Fig. S1). ResMap^42^ was used to calculate local resolutions. Resolutions are calculated according to the gold-standard FSC = 0.143 criterion^61^. The density map was corrected for the modulation transfer function (MTF) and sharpened using an automatically calculated B-factor (−104.3 Å^2^), prior to visualization.

### Model building and refinement

Coordinates for the 23S, 5S and 4.8S rRNAs were extracted from the reported coordinates of the spinach chloro-ribosome (PDB ID: 4V61)^31^. To model the missing (in PDB ID: 4V61) nucleotide residues of the 23S rRNA, sequence of the chloroplast 23S rRNA was obtained from the complete chloroplast genome sequence in NCBI (Accession no. AJ400848.1) and a homology model was generated for the complete 23S rRNA using ModeRNA server^62^. The missing nucleotide residues of the 23S rRNA were extracted from the generated homology model and connected to the 23S rRNA coordinates from PDB ID of 4V61^31^ and thereafter manually rebuilt in COOT^63^ guided by the predicted secondary structure from the Comparative RNA Website (CRW)^64^. To optimize the fitting in the density, base-pairing restraints were generated using ‘PDB to 3D Restraints’ server (http://rna.ucsc.edu/pdbrestraints/index.html) and used throughout the refinement cycles of the rRNA models using *phenix.real_space_refine*^65^.

To model chloro-ribosome LSU proteins, sequence information for the mature proteins were obtained from UniProt^66^ and GenBank^67^. Protein coordinates isolated from recently solved high resolution structure of the *E. coli* ribosome (PDB ID: 4YBB)^43^ were used as templates to guide homology modelling using ITASSER server^68^. Homology models were rigid body fitted into their respective densities (guided by positioning of the proteins in PDB ID: 4YBB) using ‘Fit in Map’ functionality in UCSF Chimera^69^ and were manually adjusted in COOT^63^, to achieve optimal fitting. Most of the chloroplast-specific extensions of the chloro-ribosome proteins didn’t fit into the density and were deleted from the homology models. These extensions were manually built using a combination of ‘C-alpha baton mode’ and ‘add terminal residue’ functionalities in COOT^63^. Extensions which assumed well defined helical structures, were *de novo* modelled using the tool ‘place helix here’ in COOT^63^. In all stages of modelling, ‘Real Space Refine Zone’ and ‘Regularize Zone’ were used to locally fit model into density while maintaining the geometry. PSRP5 and PSRP6 were modelled *de novo* using the toolsets mentioned above. Optimal fitting of individual proteins in the density was achieved by running *phenix.real_space_refine*^65^ on individual protein coordinates using local rotamer fitting, morphing, simulated annealing and secondary structure restraints applied throughout the refinement cycles. At the final stage, the coordinates for the rRNAs and the proteins were merged together in COOT^63^ and *phenix.real_space_refine*^65^ was run again using default parameters with secondary structure and base pair restraints in order to improve the map fitting and avoid clashes. In spite of the high-quality of our cryo-EM map, register shifts at poorly resolved parts and peripheral regions could not be excluded.

### Model validation

The entire structure for the spinach chloro-ribosome LSU was validated using Molprobity^70^. A detailed account of model validation and refinement statistics is presented in Supplementary Table S2.

### Figures

UCSF Chimera^69^, PyMOL^71^ and COOT^63^ are used to render all the figures. The simulated path of the nascent chain is built in COOT^63^ using the cryo-EM reconstruction reported by Bhushan *et al*. (EMDB ID: 1829)^72^. Jalview^73^ is used to generate the sequence alignment for the protein L23.

## Additional Information

**Accession codes:** The 3.5 Å cryo-EM map of the spinach chloro-ribosome LSU has been deposited in the Electron Microscopy Data Bank with accession code EMD-9572, the coordinates of the atomic model have been deposited in the Protein Data Bank under accession code 5H1S.

**How to cite this article**: Ahmed, T. *et al*. Cryo-EM structure of the large subunit of the spinach chloroplast ribosome. *Sci. Rep.* **6**, 35793; doi: 10.1038/srep35793 (2016).

## Supplementary Material

Supplementary Information

## Figures and Tables

**Table 1:** Summary of the proteins present in the spinach chloro-ribosome LSU.

Protein name	Chain ID	Uniprot ID (GenBank ID)	Mature protein (range, aa)	Built residues (range, aa)	Size of homologous *E. coli* proteins (aa)	Comments
uL1c		Q9LE95	73–352		234	Poor density, model could not be built
uL2c	E	P06509	1–272	23–270	273	
uL3c	F	(KNA04906.1)	85–305	86–297	209	
uL4c	G	O49937	51–293	52–261	201	
uL5c	H	(KNA19821.1)	39–258	56–230	179	
uL6c	I	(KNA14466.1)	39–220	41–214	177	
bL9c	J	(KNA21690.1)	42–196	43–95	149	
uL10c		(KNA14105.1)	53–232		165	Poor density, model could not be built
uL11c		P31164	67–224		142	Poor density, model could not be built
bL12c		P02398	57–189		121	Poor density, model could not be built
uL13c	L	P12629	60–250	100–246	142	
uL14c	M	P09596	1–121	1–121	123	
uL15c	N	(KNA06817.1)	80–271	93–254	144	
uL16c	O	P17353	1–135	2–135	136	
bL17c	P	(KNA19692.1)	11–126	11–126	127	
uL18c	Q	(KNA08833.1)	44–166	47–166	117	
bL19c	R	P82413	78–233	118–232	115	
bL20c	S	P28803	1–128	3–117	118	
bL21c	T	P24613	56–256	91–234	103	
uL22c	U	P09594	1–199	26–149	110	
uL23c	V	Q9LWB5	77–198	105–193	100	
uL24c	W	P27683	47–191	50–173	104	
bL27c	X	(KNA14420.1)	58–194	68–165	85	
bL28c	Y	(KNA16864.1)	72–148	72–145	78	
uL29c	Z	(KNA15596.1)	60–168	63–149	63	
bL31c	a	(KNA12538.1)	37–130	38–75	70	Poor density
bL32c	b	P28804	1–57	2–47	57	
bL33c	c	P28805	1–66	9–59	55	
bL34c	d	P82244	92–152	92–148	46	
bL35c	e	P23326	87–159	91–157	65	
bL36c	f	P12230	1–37	1–37	38	
PSRP5	g	P27684	63–142	79–121		PSRP variant 5α, pI = 10.01
PSRP6	h	P82411	48–116	48–93		pI = 9.87

Protein residues and signal peptide positions are numbered according to the information present in Uniprot^66^, GenBank^67^ and coordinates from PDB ID: 4V61^31^.

Abbreviations: PSRP = Plastid-specific ribosomal protein.
