# Supplementary material for: Cryo-EM structure of the large subunit of the spinach chloroplast ribosome
Source: Sci Rep. 2016 Oct 20;6:35793. doi: 10.1038/srep35793 (PMC5071890; doi:10.1038/srep35793)
Supplement: Supplementary Information [file srep35793-s1.pdf]

SUPPLEMENTARY INFORMATION

Cryo-EM structure of the large subunit of the spinach chloroplast ribosome

Tofayel Ahmed[1], Zhan Yin[1] and Shashi Bhushan[1,2,*]

[1]School of Biological Sciences, Nanyang Technological University, Singapore

[2]NTU Institute of Structural Biology, Nanyang Technological University, Singapore

* To whom correspondence should be addressed. Tel: +65 6592 3673; Fax: +65 6791 3856;

E-mail: sbhushan@ntu.edu.sg

Figure S1

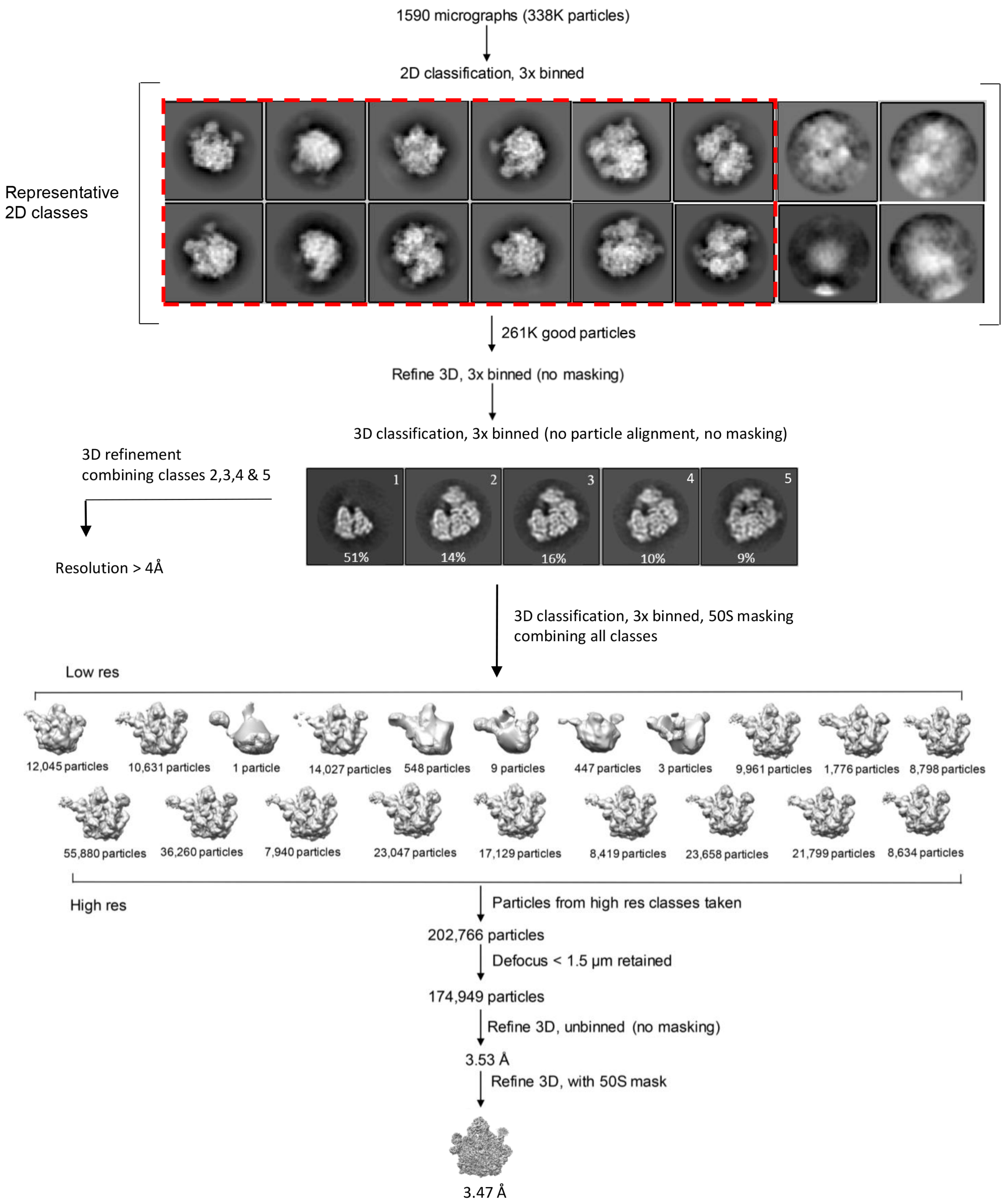

1590 micrographs (338K particles)
2D classification, 3x binned
Representative 2D classes
261K good particles
Refine 3D, 3x binned (no masking)
3D classification, 3x binned (no particle alignment, no masking)
3D refinement combining classes 2,3,4 & 5
Resolution > 4Å
1
2
3
4
5
51%
14%
16%
10%
9%
3D classification, 3x binned, 50S masking combining all classes
Low res
12,045 particles
10,631 particles
1 particle
14,027 particles
548 particles
9 particles
447 particles
3 particles
9,961 particles
1,776 particles
8,798 particles
55,880 particles
36,260 particles
7,940 particles
23,047 particles
17,129 particles
8,419 particles
23,658 particles
21,799 particles
8,634 particles
High res
Particles from high res classes taken
202,766 particles
Defocus < 1.5 µm retained
174,949 particles
Refine 3D, unbinned (no masking)
3.53 Å
Refine 3D, with 50S mask
3.47 Å

Figure S2

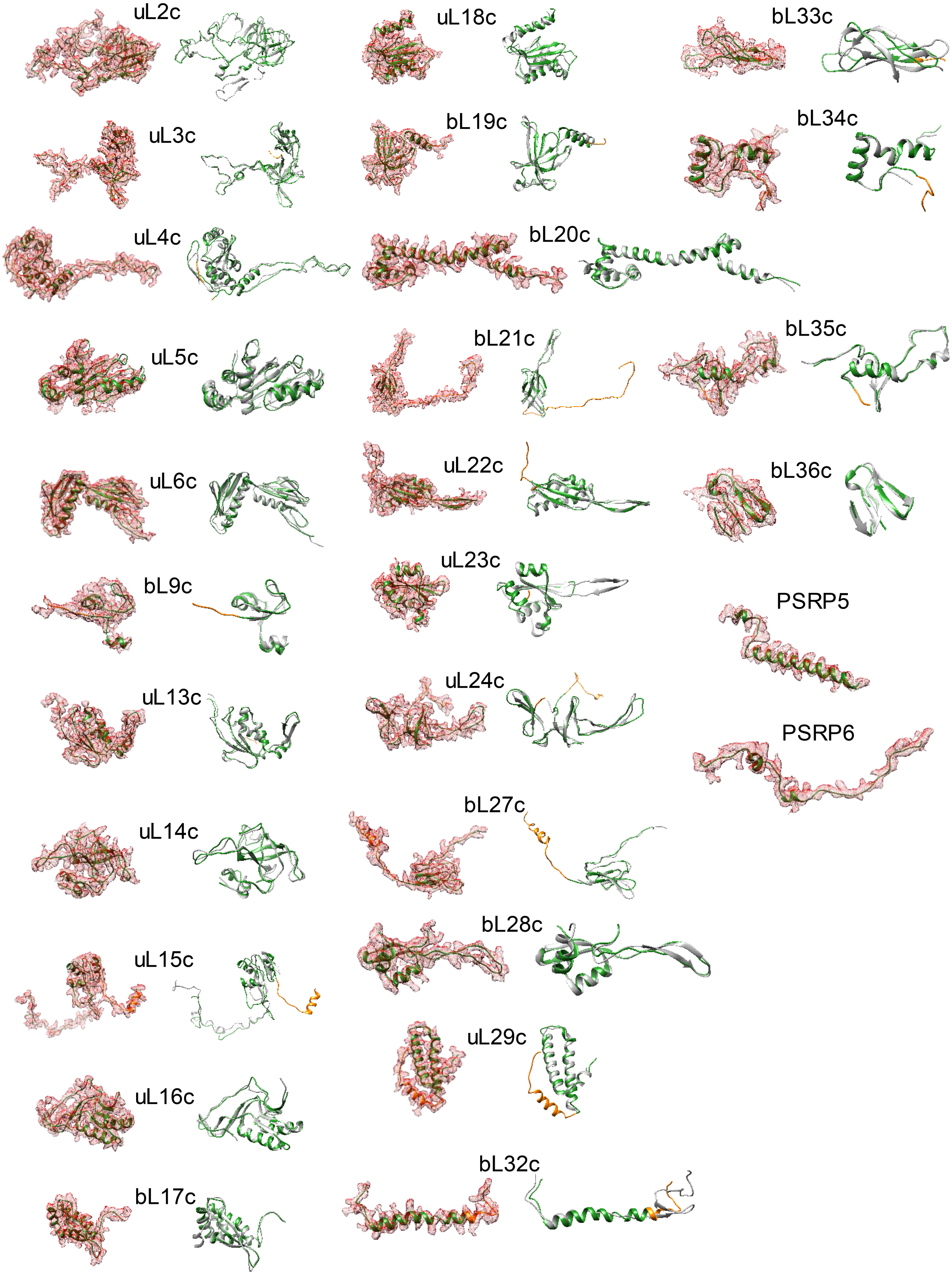
uL2c
uL18c
bL33c
uL3c
bL19c
bL34c
uL4c
bL20c
uL5c
bL21c
bL35c
uL6c
uL22c
bL36c
bL9c
uL23c
PSRP5
uL13c
uL24c
PSRP6
uL14c
bL27c
uL15c
bL28c
uL29c
uL16c
bL17c
bL32c

Figure S3

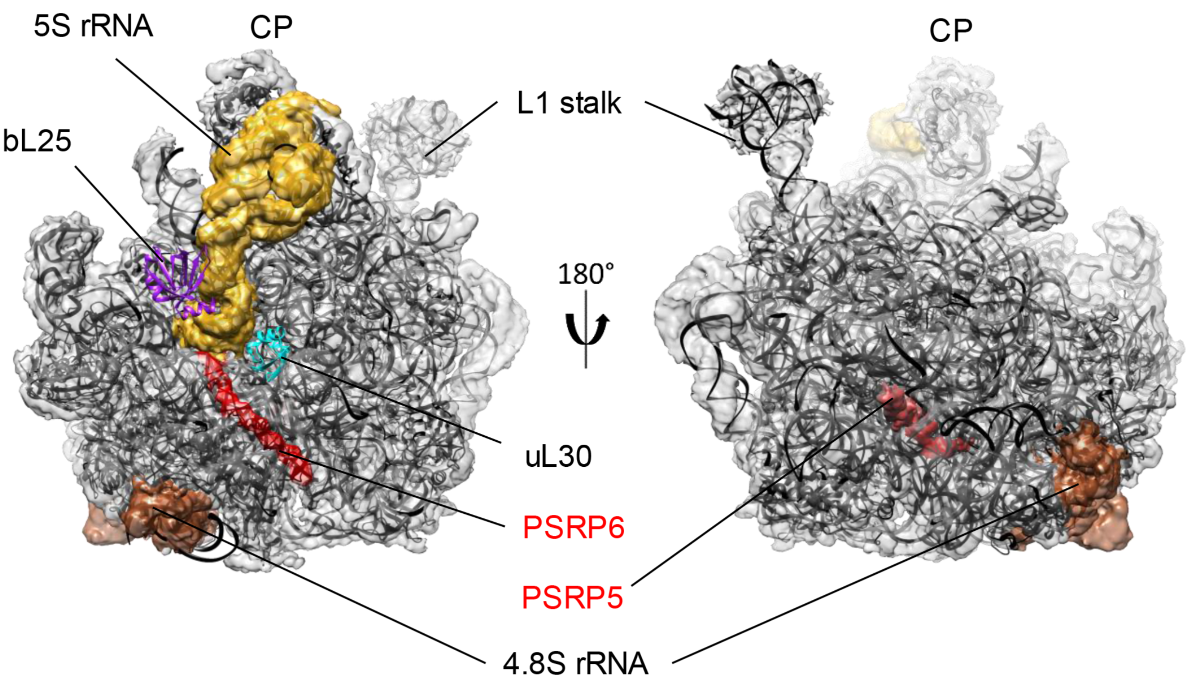

Figure S4

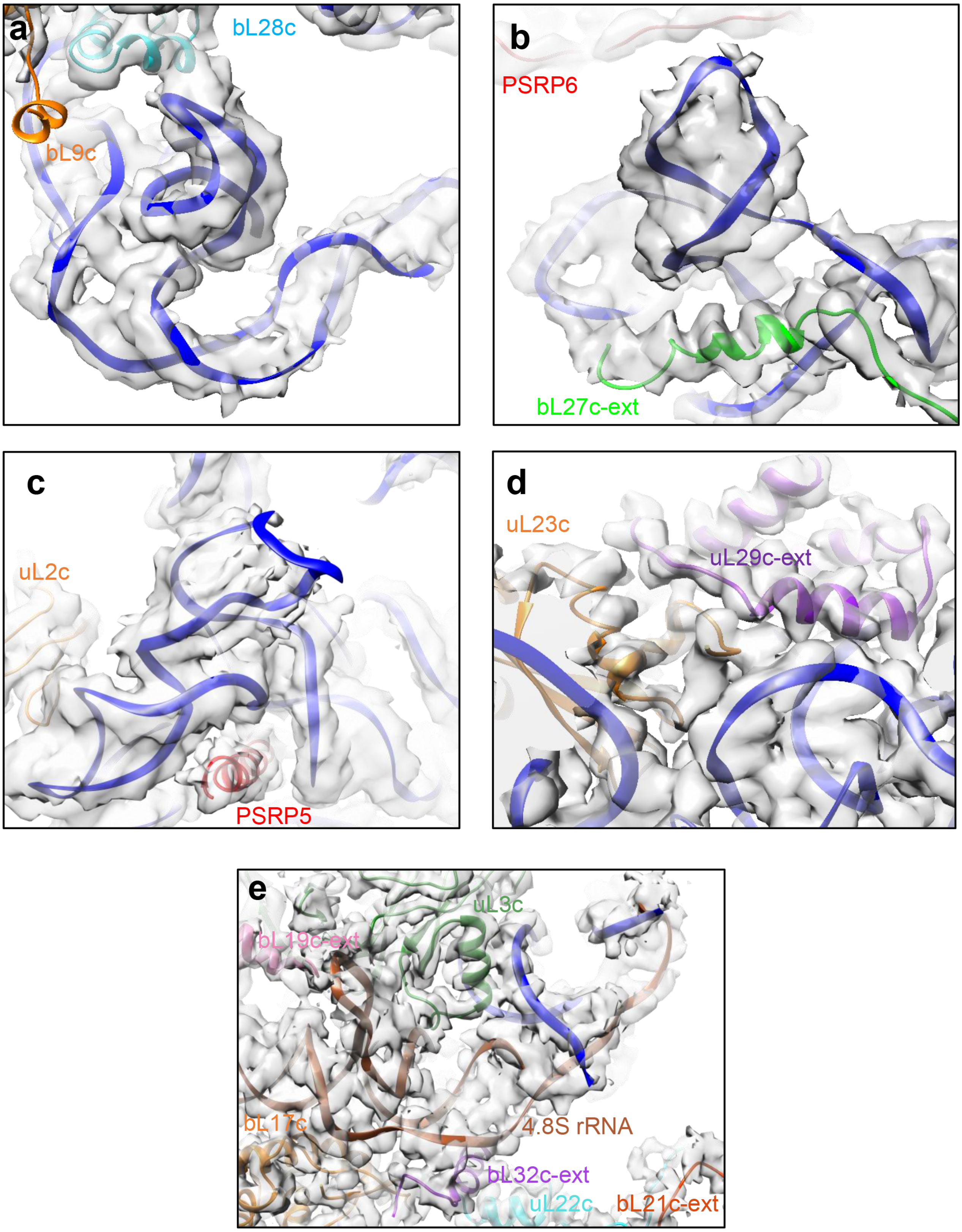
a
bL28c
bL9c
b
PSRP6
bL27c-ext
c
uL2c
PSRP5
d
uL23c
uL29c-ext
e
uL3c
bL19c-ext
bL17c
4.8S rRNA
bL32c-ext
uL22c
bL21c-ext

Figure S5

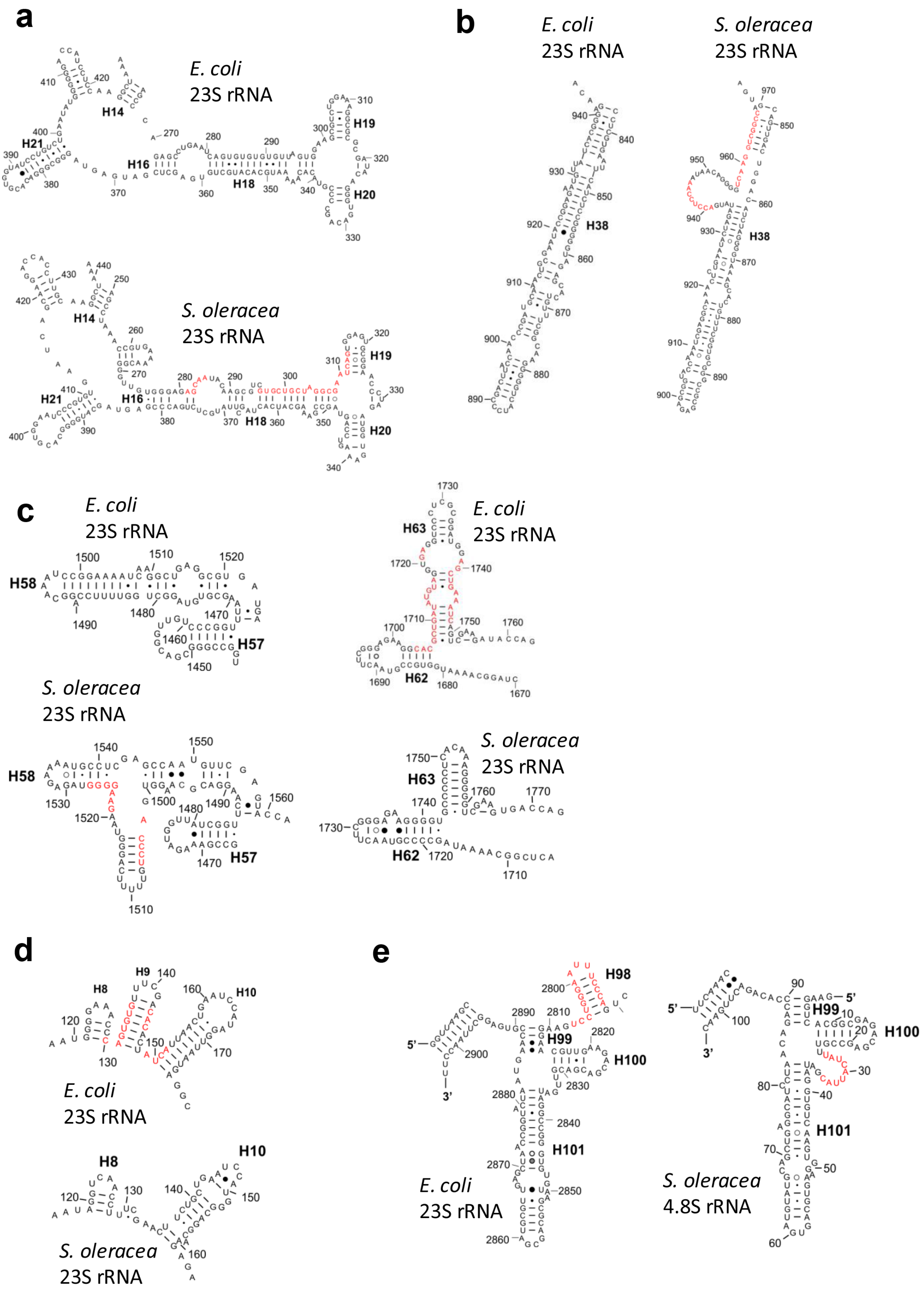
a
E. coli
23S rRNA
H14
H21
H16
H18
H19
H20
S. oleracea
23S rRNA
b
E. coli
23S rRNA
S. oleracea
23S rRNA
H38
c
E. coli
23S rRNA
H58
H57
H63
H62
S. oleracea
23S rRNA
d
H8
H9
H10
E. coli
23S rRNA
S. oleracea
23S rRNA
e
H98
H99
H100
H101
E. coli
23S rRNA
S. oleracea
4.8S rRNA

Figure S6

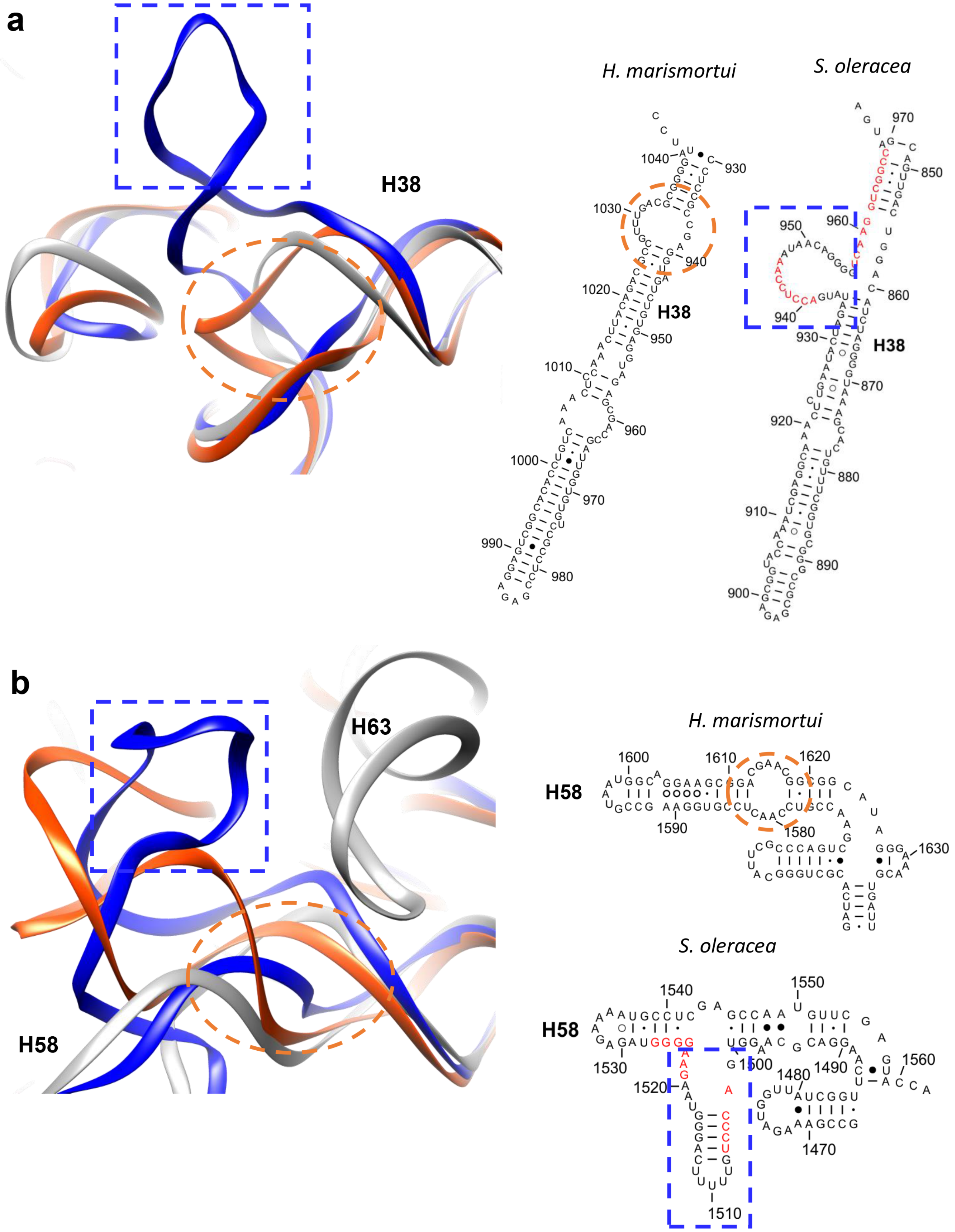
a
H38
H. marismortui
S. oleracea
H38
H38
b
H63
H58
H. marismortui
H58
S. oleracea
H58

Figure S7

a

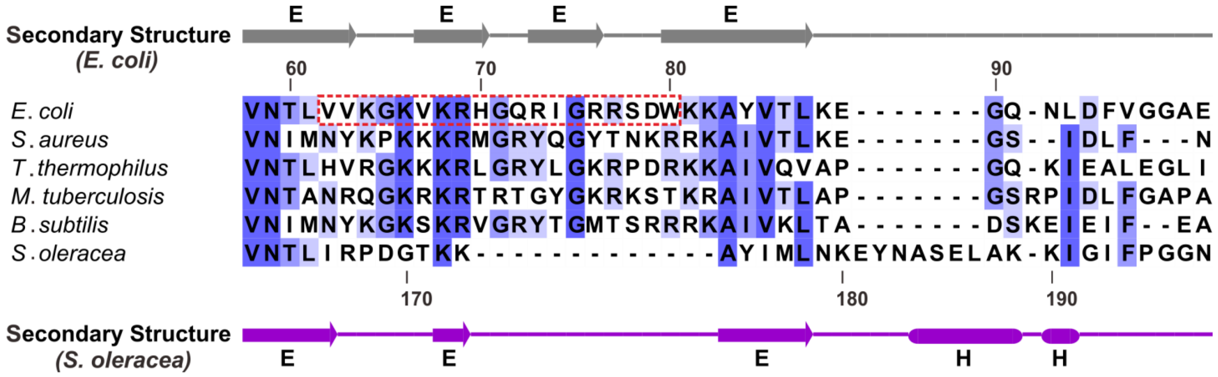
Secondary Structure
(E. coli)
E
60
70
80
90
E. coli
S. aureus
T. thermophilus
M. tuberculosis
B. subtilis
S. oleracea
VNTLVVKGKVKRHGQRIGRRSDWKKAYVTLKE-------GQ-NLDFVGGAE
VNIMNYKPKKKRMGRYQGYTNKRRKAIVTLKE-------GS--IDLF---N
VNTLHVRGKKKRLGRYLGKRPDRKKAIVQVAP-------GQ-KIEALEGLI
VNTANRQGKRKRTRTGYGKRKSTKRAIVTLAP-------GSRPIDLFGAPA
VNIMNYKGKSKRVGRYTGMTSRRRKAIVKLTA-------DSKEIEIF--EA
VNTLIRPDGTKK-------------AYIMLNKEYNASELAK-KIGIFPGGN
170
180
190
Secondary Structure
(S. oleracea)
H

b

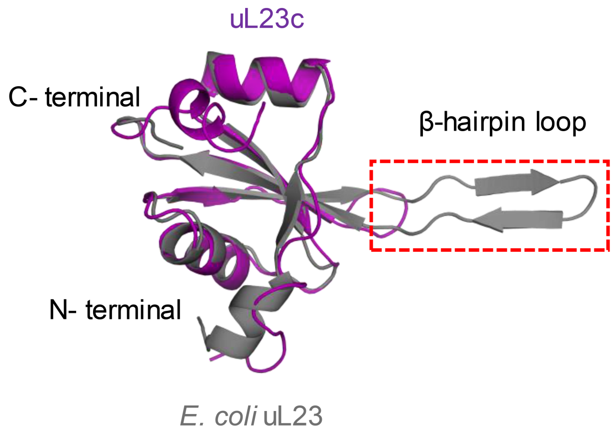
uL23c
C- terminal
β-hairpin loop
N- terminal
E. coli uL23

SUPPLEMENTARY FIGURE LEGENDS

Figure S1:

Cryo-EM data processing. From 1590 micrographs collected, a total of 338K particles were picked semi-automatically using e2boxer.py tool from EMAN 2.1[1]. Data were processed using RELION1.4[2]. 3X binned particles were sorted using 2D classification step, discarding 77K particles. Thereafter, 3D refinement was used to assign the correct angles for the particles and 3D classification was carried out to sort particles in 50S and 70S classes. Particles from 70S classes did not yield a high-resolution map suitable for modelling. Combined 50S and 70S particles were further subjected to 3D classification using a 50S mask to discard low-resolution particles. For subsequent processing, particles from high defocus micrographs (>1.5 µm) were discarded and 3D refinement was performed with unbinned particles (without masking) resulting in a density map resolved to 3.53 Å. Final 3D refinement applying a 50S mask improved resolution to 3.47 Å.

Figure S2:

Spinach chloro-ribosome LSU proteins fitted into respective density. Spinach chloro-ribosome LSU protein models rigid-body fitted into their respective density (left) isolated from the chloro-ribosome 50S cryo-EM map; homologous *E. coli* protein models (grey) are overlaid with chloro-ribosome LSU proteins (forest green) to show comparison. Chloroplast specific protein extensions are coloured orange. uL1c, uL10c, uL11c and bL12c could not be modelled due to poorly resolved density. EM density is shown in transparent mesh and coloured red. Volume Viewer-> Features-> Zone functionality from UCSF Chimera[3] was used to isolate the density for the protein models.

Figure S3:

Absence of bL25 and uL30 in the spinach chloro-ribosome LSU. *E. coli* 50S model (PDB ID: 4YBB) rigid body fitted into the cryo-EM map of the spinach chloro-ribosome LSU. Most parts fit well except bL25 and uL30 denoting the absence of these two proteins in the spinach chloro-ribosome LSU. The density for the central protuberance (CP), L1 stalk, 5S rRNA (golden) and 4.8S rRNA (sienna), PSRP5 (red) and PSRP6 (red) are indicated. Models for the rRNAs and the proteins except bL25 and uL30 are coloured black. The chloro-ribosome LSU density is low-pass filtered and shown in transparent surface.

Figure S4:

Density showing remodelled rRNAs of the spinach chloro-ribosome LSU. Density for the remodelled rRNA structures of the spinach chloro-ribosome LSU are shown. Models for 23S rRNA of the spinach chloro-ribosome LSU is colored in blue. Chloro-ribosome LSU proteins and 4.8S rRNA are labelled as indicated. In all cases, the density is filtered and shown in transparent surface.

Figure S5:

Secondary structure of remodelled spinach chloro-ribosome LSU rRNAs. Secondary structures of *E. coli* and spinach chloro-ribosome LSU rRNAs are compared. Insertions and deletions are indicated in red. The secondary structures are obtained from Comparative RNA Website[4] and chloro-ribosome LSU rRNA secondary structure is modified according to the information obtained from the models generated.

Figure S6:

Introduction of protrusions in chloro-ribosome 23S rRNA near K-turn structures present in H. marismortui 23S rRNA. 23S rRNAs of *H. marismortui* (orange) and spinach

chloro-ribosome (blue) are overlaid to show presence of protrusions (shown using blue rectangles) in the chloro-ribosome 23S rRNA near to sites where K-turn structures (shown using orange circles) are present in *H. marismortui* rRNA[5]. *E. coli* 23S rRNA (grey) is shown for comparison. Insertions in chloro-ribosome sequence are marked in red. The *H. marismortui* structure is obtained from PDB ID: 1FFK[6].

Figure S7:

Absence of β-hairpin loop in uL23c. a. Multiple sequence alignment of the protein L23 sequences from different bacterial species and spinach chloroplast showing absence of partially conserved β-hairpin loop in chloro-ribosome. Alignment is generated using T-Coffee server[7] and secondary structures are derived directly from the structures using Jalview[8]. b. Coordinates of *E. coli* L23 from PDB ID: 4YBB overlaid with the coordinates of chloro-ribosome L23 showing the absence of the β-hairpin loop in chloro-ribosome.

Table S1 Chloroplast-specific protein extensions in the spinach chloro-ribosome LSU. Length of the chloroplast-specific protein extensions present in the *E. coli* homologous proteins are indicated along with the lengths modelled in the current study.

Protein name	N-terminal extension length (aa)	N-terminal extension modelled (aa)	C-terminal extension length (aa)	C-terminal extension modelled (aa)
uL1c	42	0	4	0
uL2c	0	0	0	0
uL3c	4	3	7	0
uL4c	6	5	33	1
uL5c	15	0	26	0
uL6c	1	0	3	0
bL9c	7	6	0	0
uL10c	0	0	14	0
uL11c	5	0	12	0
bL12c	4	0	0	0
uL13c	40	0	9	5
uL14c	0	0	0	0
uL15c	0	0	37	20
uL16c	0	0	0	0
bL17c	0	0	0	0
uL18c	3	0	0	0
bL19c	46	6	0	0
bL20c	0	0	4	0
bL21c	67	32	30	8
uL22c	29	4	60	12
uL23c	28	0	7	2
uL24c	18	15	22	4
bL27c	0	0	53	24
bL28c	0	0	0	0
uL29c	3	0	43	24
bL31c	0	0	23	0
bL32c	0	0	23	13
bL33c	11	4	0	0
bL34c	0	0	10	8
bL35c	2	0	6	4
bL36c	0	0	0	0

Table S2 Refinement and model statistics for the spinach chloro-ribosome LSU. The spinach chloro-ribosome LSU model was refined against the 3D reconstruction of LSU generated after the masked refinement.

**Data Collection**	
Particles	338,305
Pixel Size (Å)	1.28
Defocus Range (μm)	0.2-2.5
Voltage (kV)	200
Electron Dose ($e^{-}Å^{-2}$)	26

**Model composition**	
Non-hydrogen atoms	90,671
Protein residues	3,229
RNA bases	3,029

**Refinement**	
Resolution (Å)	3.47
Map sharpening B-factor ($Å^2$)	-104.3
CC_{map_model}	0.72

**RMS deviation**	
Bonds (Å)	0.01
Angles (°)	1.23

**Validation (proteins)**	
Molprobity score	2.65 (39th percentile)
Clashscore, all atoms	25.65 (20th percentile)
Poor rotamers (%)	0.51
Favoured rotamers (%)	97.15

**Ramachandran plot**	
Favoured (%)	78.87
Outliers (%)	0.35

**Validation (RNA)**	
Correct sugar puckers (%)	95.18
Good backbone conformations (%)	60.32

References

1 Bell, J. M., Chen, M., Baldwin, P. R. & Ludtke, S. J. High resolution single particle refinement in EMAN2.1. *Methods* **100**, 25-34, (2016).

2 Scheres, S. H. W. RELION: Implementation of a Bayesian approach to cryo-EM structure determination. *Journal of Structural Biology* **180**, 519-530, (2012).

3 Pettersen, E. F. *et al.* UCSF Chimera—A visualization system for exploratory research and analysis. *Journal of Computational Chemistry* **25**, 1605-1612, (2004).

4 Cannone, J. J. *et al.* The comparative RNA web (CRW) site: an online database of comparative sequence and structure information for ribosomal, intron, and other RNAs. *BMC Bioinformatics* **3**, 2, (2002).

5 Klein, D. J., Schmeing, T. M., Moore, P. B. & Steitz, T. A. The kink-turn: a new RNA secondary structure motif. *EMBO J* **20**, 4214-4221, (2001).

6 Ban, N., Nissen, P., Hansen, J., Moore, P. B. & Steitz, T. A. The Complete Atomic Structure of the Large Ribosomal Subunit at 2.4 Å Resolution. *Science* **289**, 905-920, (2000).

7 Di Tommaso, P. *et al.* T-Coffee: a web server for the multiple sequence alignment of protein and RNA sequences using structural information and homology extension. *Nucleic Acids Research* **39**, W13-W17, (2011).

8 Waterhouse, A. M., Procter, J. B., Martin, D. M. A., Clamp, M. & Barton, G. J. Jalview Version 2—a multiple sequence alignment editor and analysis workbench. *Bioinformatics* **25**, 1189-1191, (2009).
